# *Cryptosporidium*, *Giardia*, *Cryptococcus*, *Pneumocystis* Genetic Variability: Cryptic Biological Species or Clonal Near-Clades?

**DOI:** 10.1371/journal.ppat.1003908

**Published:** 2014-04-10

**Authors:** Michel Tibayrenc, Francisco J. Ayala

**Affiliations:** 1 Maladies Infectieuses et Vecteurs Ecologie, Génétique, Evolution et Contrôle, MIVEGEC (IRD 224-CNRS 5290-UM1-UM2), IRD Center, BP 64501, Montpellier, France; 2 Department of Ecology and Evolutionary Biology, University of California, Irvine, California, United States of America; The Fox Chase Cancer Center, United States of America

## Abstract

An abundant literature dealing with the population genetics and taxonomy of *Giardia duodenalis*, *Cryptosporidium* spp., *Pneumocystis* spp., and *Cryptococcus* spp., pathogens of high medical and veterinary relevance, has been produced in recent years. We have analyzed these data in the light of new population genetic concepts dealing with predominant clonal evolution (PCE) recently proposed by us. In spite of the considerable phylogenetic diversity that exists among these pathogens, we have found striking similarities among them. The two main PCE features described by us, namely highly significant linkage disequilibrium and near-clading (stable phylogenetic clustering clouded by occasional recombination), are clearly observed in *Cryptococcus* and *Giardia*, and more limited indication of them is also present in *Cryptosporidium* and *Pneumocystis*. Moreover, in several cases, these features still obtain when the near-clades that subdivide the species are analyzed separately (“Russian doll pattern”). Lastly, several sets of data undermine the notion that certain microbes form clonal lineages simply owing to a lack of opportunity to outcross due to low transmission rates leading to lack of multiclonal infections (“starving sex hypothesis”). We propose that the divergent taxonomic and population genetic inferences advanced by various authors about these pathogens may not correspond to true evolutionary differences and could be, rather, the reflection of idiosyncratic practices among compartmentalized scientific communities. The PCE model provides an opportunity to revise the taxonomy and applied research dealing with these pathogens and others, such as viruses, bacteria, parasitic protozoa, and fungi.

## Author Summary

Micropathogen species definition is extremely difficult, since concepts applied to higher organisms (the biological species concept) are inadequate. In particular, the pathogens here surveyed have given rise to long-lasting controversies about their species status and that of the genotypes that subdivide them. The population genetic approach based on the predominant clonal evolution (PCE) concept proposed by us could bring simple solutions to these controversies, since it permits the description of clearly defined evolutionary entities (clonal multilocus genotypes and near-clades [incompletely isolated clades]) that could be the basis for species description, if the concerned specialists find it justified for applied research. The PCE model also provides a convenient framework for applied studies (molecular epidemiology, vaccine and drug design, clinical research) dealing with these pathogens and others.

## Introduction: The Model of Predominant Clonal Evolution (PCE)

The PCE model [1], [2] defines clonal evolution as scarcity or absence of genetic recombination, a definition that is accepted by most authors working on pathogen population genetics [3], including the species here surveyed [4]–[9]. The PCE model [3], [10], [11] (i) does not presume that recombination is absent [12], [13] or plays a minor evolutionary role, but that it is too rare to break the prevalent pattern of clonality; (ii) addresses each species as a whole, and not their genetic subdivisions considered individually [14]; and (iii) definitely includes selfing/inbreeding/homogamy (which lead to restrained recombination) as particular cases of PCE, rather than as distinct evolutionary models [1]–[3], [10], [11], [15]. This view is shared by many authors working on the pathogens here analyzed [12], [16]–[19] and by others [20]. A few authors [21], [22] prefer to limit the concept of clonality to “strict” clonality (i.e., mitotic propagation) and consider that it should be distinguished from selfing/inbreeding/“unisex.” This is a matter of definition. It is nevertheless worth noting that in the examples cited in [21], differently from the authors of the article, all scientists working on parthenogenesis in insects, amphibians, fishes, and reptiles definitely include parthenogenesis in clonality.

As we have exposed extensively [1]–[3], biases that could lead to wrong conclusions of restrained recombination (mainly isolation by distance and/or time or Wahlund effect) should be carefully considered before concluding a PCE pattern.

Lastly, as we have insisted in [3], the PCE model states that restrained recombination is mainly due to built-in properties of microbes, rather than to the downstream elimination of most possible recombinants by natural selection and epistasis phenomena. If natural selection were the main factor that would maintain clonality, it would be at unacceptable costs for the organisms considered, because this would mean that most of the offspring is eliminated at each generation. Natural selection certainly acts on microbes, as it does on any organism. However, our proposal is that it cannot be the main factor responsible for PCE in organisms that would be otherwise potentially panmictic.

## Recent Developments

We have recently proposed new insights about PCE, applicable to all kinds of micropathogens (including viruses, bacteria, parasites, and fungi) [3] and, more specifically, to *Trypanosoma* and *Leishmania* [10] and to *Plasmodium* and *Toxoplasma* [11]. We have proposed replacing subjective and imprecise assertions such as “recombination at a high rate” [14] or “gross incongruences” [23] with a clear-cut PCE definition relying on two complementary criteria: (i) statistically significant linkage disequilibrium (LD), or nonrandom association of genotypes occurring at different loci, and (ii) growing phylogenetic signal when more reliable data are added. Lastly, we have discussed the possibility of distinguishing PCE from cryptic biological speciation. We have also distinguished clonality by lack of available mating partners (due to scarcity of multiclonal infections) from built-in clonality.

LD is the very statistic that permits one to evidence lack of recombination, the basic definition of PCE. Contrary to segregation tests, LD analysis does not require that the organism under survey is diploid, nor does it require knowledge of ploidy [3]. This is highly relevant when micropathogens are concerned [3] since widespread aneuploidy seems to be very frequent in them, including in fungi, *Trypanosoma*, and *Leishmania* [12], which renders tests based on diploidy invalid. When a sufficient set of loci is analyzed, LD is a very powerful statistic [1].

One has to ascertain that LD cannot be explained by trivial physical obstacles (isolation by space or time: the Wahlund effect) [2]. It is widely used as circumstantial evidence for PCE by authors working on the pathogens here considered [7], [24]–[26] and by others [27], [28]. A telling consequence of LD is the spread of stable multilocus genotypes (MLGs) over vast time and space scales [3]. However, this pattern depends on the rate of evolution (molecular clock) of the marker considered and might not be observed with fast-evolving markers such as microsatellites, even in the case of strong linkage disequilibrium [3].

The criterion of a growing phylogenetic signal when more adequate data are added relies on the congruence principle [29], which states that if the working hypothesis is correct, evidence increases as more data are considered. For example, when a set of Multilocus Sequence Typing (MLST) data are considered, although some discrepancies can be observed between individual gene trees, the phylogenetic signal gets stronger and stronger when more loci are included in the combined tree. Or, the genetic distances calculated from different molecular markers are strongly correlated (the “g” test [1]). If the impact of recombination were stronger than clonal propagation in the long run, the contrary would obtain. This approach, relying on congruence, may not be verified when inadequate data are compared, such as, for example, markers with different molecular clocks or undergoing different selective pressures or different evolutionary tendencies. This could lead to wrong assertions of recombination [10]. The main manifestation of this growing phylogenetic signal is the existence of genetic subdivisions that are stable in space and time (“near-clades” [3]). The term “clade” [26], [30], [31] is not adequate when micropathogens are concerned, because even when PCE obtains, some residual recombination can always occur [3].

**Figure 1:**
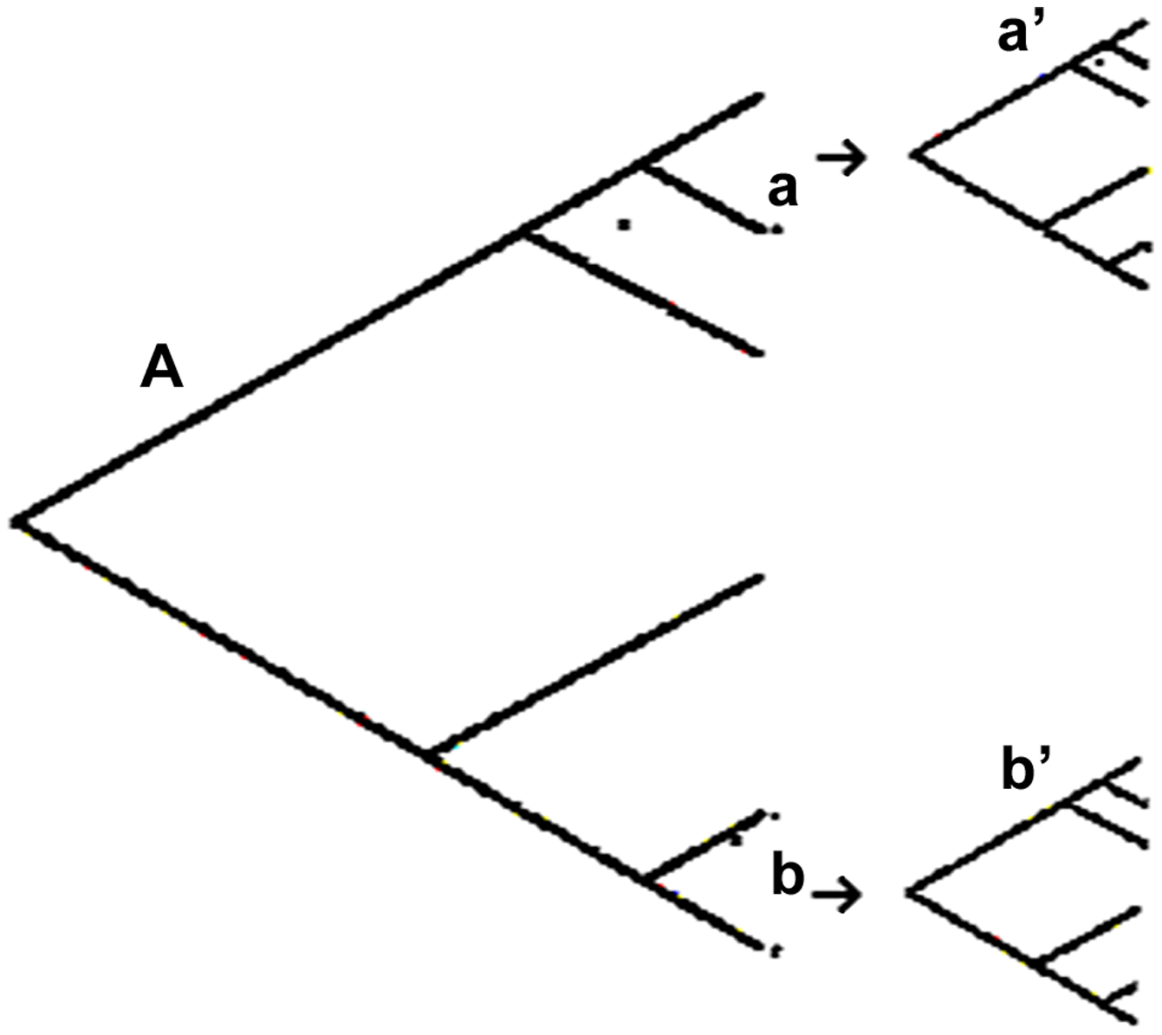
“Russian doll” model [10]. When population genetic tests are performed with appropriate markers (of sufficient resolution) within each of the near-clades, a and b, that subdivide the species, A, under study (large tree, left part of the figure), they reveal within these near-clades a miniature picture of the whole species, with the two main PCE features, namely linkage LD and lesser near-clades (two small trees, a′ and b′, right part of the figure). This shows that PCE obtains also within the near-clades, and that these do not correspond to cryptic, potentially panmictic, biological species.

We have differentiated PCE from cryptic speciation. It has been inferred that apparent clonality could be explained by the fact that the species under study is subdivided into discrete genetic clusters, among which recombination is inhibited while it is not within them [32]. Such a model amounts to equating these genetic subdivisions to cryptic biological species. To distinguish this case from PCE, we have proposed [10] the “Russian doll model.” If the PCE criteria are uncovered, not only at the level of the whole species but also within its genetic subdivisions, it favors PCE rather than cryptic speciation. In this case, the genetic subdivisions of the species show a miniature picture of the whole species, with LD and lesser near-clades (Figure 1). However, this approach should be conveniently applied by selecting markers with an adequate resolution power (molecular clock). As a matter of fact, when addressing lesser genetic subdivisions rather than the whole species, one changes evolutionary scales. If the resolution of the markers is not consequently adapted, lack of PCE signal could be due to a statistical type II error (lack of resolution). For the same reason, the sampling size should not become too small.

We have also discussed apparent clonality by lack of available mating partners in low transmission cycles. To explain apparent manifestations of clonality in *Plasmodium falciparum* [1], [2], it has been proposed that selfing/inbreeding occurred “mechanically” in low transmission areas because mixed infections of different genotypes are rare, which makes outcrossing impossible [33]. We have called this model the “starving sex hypothesis” and have shown that it was frequently at odds with the available data in *P. falciparum* as well as in *P. vivax* [11]. The alternative hypothesis [11] is that restrained recombination by selfing, inbreeding, or any other mechanism, is a built-in evolutionary strategy used by the pathogen to avoid the “recombinational load” (break-up of favorable MLGs by recombination [34]), even when different MLGs are available for mating. Inbreeding/selfing, unisexual reproduction can be considered as a way to add limited phenotypic and genotypic diversity in a clonal population without breaking favorable multilocus combinations [12], [18]. *Cryptococcus* and *Giardia* possess meiosis genes [17], [35]. However, these genes could be associated with other functions than meiosis: “Evolution is constantly re-using old genes for new purposes” [16]. We have proposed [3] that many micropathogens could possess a “clonality/sexuality machinery” rather than meiosis genes for switching between clonal evolution and recombination to face various evolutionary challenges. Selfing could be used by them instead of outcrossing, even when mating partners are available.

## PCE Manifestations in the Pathogens under Survey

We have proposed [1], [2] that *Giardia duodenalis* and *Cryptococcus neoformans* undergo PCE. Contrary to *Plasmodium* [1], [11], this proposal did not lead to hot controversy. That clonality is strong or preponderant is accepted in *Cryptococcus* [5], [17], [32] and *G. duodenalis* [4], [36], [37] and has been proposed for *Cryptosporidium hominis* [7]. As a matter of fact, the main PCE manifestations are easily observable in these pathogens. A few examples among the many available include the following:

- LD: It has been recorded in *C. gattii* [30], [38]–[40], *C. neoformans* [26], [30], [38], [40], [41], *Pneumocystis jirovecii* [25], *Cr. hominis* [7] and *G. duodenalis* [13], [37].
- Widespread, stable MLGs: In *C. gatti*, the MLG responsible for the “Vancouver epidemics,” sequence type (ST) 39 has been isolated in Vancouver, the United States Pacific Coast, and Korea, in humans and in animals [39], [42]. It is identical to the NIH 444 strain, isolated in 1970 [43]. In *C. neoformans* var. *grubii*, the MLG ST4 has been isolated from 1996 to 2007 in six different countries in Africa and Asia. ST5 has been isolated from 1983 to 2009 in four countries in North and South America, Europe, and Asia [26]. The MLG M5 is distributed in North and South America, Asia, Europe, and Africa [44]. In *Pn. jirovecii*, identical MLGs have been isolated in ten different European hospitals over 9 years, and in the same patients over 8 weeks [45].
- Near-clading: Near-clades are clearly identifiable in *G. duodenalis* [24], [36], [46], [47]. As a matter of fact, the *Giardia* “assemblages” are perfectly equivalent to near-clades. They are stable, widespread, and occur in sympatry, including in the same host [36]. As we have stated [3], [10], [11], the near-clades are not defined by strict phylogenetic congruence among loci, but rather, by a clear increasing phylogenetic signal when more loci are added. This is the case for *Giardia* assemblages, even if some discrepancies are observed among loci [48]. We have already called attention [3] to the fact that the many terms used by various authors to designate pathogen subspecific genetic subdivisions do not correspond to true different evolutionary entities and are rather a manifestation of the compartmentalization in this scientific milieu. We propose that the “assemblages,” “clusters,” “clonal groups,” and many other terms (see Table 1) correspond to a unique evolutionary entity, the near-clade. Using this only term instead of the many other ones that are now used in this field (see Table 1) has two main advantages: (i) the term near-clading has a clear evolutionary definition and (ii) the same evolutionary entity should not de designated by a wealth of different, imprecise terms. Obviously, this field of research calls for urgent semantic simplification. Near-clades are identified in *Cr. hominis* [7]. In the “*C. neoformans* complex of species” (CNC), the “molecular types” in *C. neoformans* VN I–IV and *C. gattii* VG I–IV [30]–[32] correspond to clearly delimited near-clades. The former species *Pn. carinii* proved to be subdivided into clearly-differentiated genotypes with strong host specificity [49], [50]. These host-specific genotypes have been given the species status, although (i) host specificity is far from absolute and (ii) indications of hybridization are recorded among them [50]. Since some indications for clonality are recorded within these genotypes [25], [45], they might be as well considered as mere near-clades.
- Russian doll patterns: In *C. gattii*, within the cluster (near-clade) VGI, clonality obtains, and four lesser subdivisions, namely C1–4, are observed [32]. In VG II, three “clonal groups,” a, b, and c, are evidenced [31], [42]. In *G. duodenalis*, assemblage A shows clear subdivisions (“subassemblages”); assemblage B and other assemblages may also exhibit subdivisions, although they are less ascertained [13], [24], [47], [51], [52].
- Data congruence: In the CNC, the near-clades are corroborated by Amplified Fragment Length Polymorphism (AFLP), MLST, PCR fingerprinting, Random Amplified Polymorphic DNA (RAPD), and Restriction Fragment Length Polymorphism (RFLP) [5], [8], [38]. The *Giardia* assemblages and their subdivisions (Russian dolls) are corroborated by Multilocus Enzyme Electrophoresis (MLEE) and sequence data [36], [47].

**Table 1:** The many different terms used in the pathogen population genetic literature to designate the same evolutionary entity (near-clade).

Viruses	Bacteria	Parasitic protozoa	Fungi
clades	clades	assemblages	AFLP groups
clusters	clonal complexes	clades	clades
genogroups	clonal lineages	clonal lineages	clonal lineages
genotypes	clusters	clones	clusters
major genotypes	genetic groups	clusters	clonal groups
major lineages	genoclouds	core subgroups	genetically distinct subgroups
phylogenetic groups	groups	discrete typing units (DTUs)	genotypes
	lineages	genetic groups	genotypic groups
		genotypes	groups
		groups	lineages
		haplotypes	molecular genotypes
		lesser subgroups	molecular types
		populations	phylogenetic species
		subassemblages	subclusters
		subgroups	subgenotypes
		subpopulations	subgroups
		subtypes	subpopulations
		subtype groups	varieties
		types	

### Starving sex versus built-in restrained recombination

Clonality in *Cryptosporidium*, whose cycle includes meiosis, is generally considered explainable by lack of outcrossing opportunity due to low transmission, or starving sex [53]. However, some data do not rule out the alternative hypothesis of built-in restrained recombination, even if the data are less conclusive than for *Plasmodium* [11]. In Ireland, *Cr. parvum* is considered panmictic due to high transmission rates. However, the percentage of multiclonal infections is lower in Ireland than in other European countries such as Italy, where *Cr. parvum* is not panmictic [54]. In the US Midwest, *Cr. parvum* is overall panmictic. However, it is “epidemic” (unstable clonality [27]) in Minnesota, where the transmission is high [55]. The *C. gatti* widespread genotype responsible for the Vancouver epidemics is supposed to be the result of “same sex mating” between identical MLGs [38]. This results in “meiotically-derived clones undetectable by molecular approaches” [43]. However, it cannot be inferred from the data whether same-sex mating is the result of starving sex or of built-in restrained recombination.

In summary, evidence that the main PCE signs obtain is strong in *G. duodenalis* and the CNC. Both present striking similarities with many other pathogens, for example, *Trypanosoma cruzi* [10] and *Toxoplasma gondii* [11], [56], with significant LD; clearly delimited near-clades; ubiquitous, stable MLGs; and “Russian doll” patterns within the near-clades. Both *Giardia* and the CNC also present indications for limited recombination or hybridization, both within and between near-clades [36], [47], [57], and even between species in the case of the CNC [41]. As is the case for *T. cruzi* [58] and *Toxoplasma* [56], patterns of hybridization might be complex [41]. The case of *Cryptosporidium* is less clear. This apicomplexa genus is known to undergo a sexual phase during transmission cycles, as do *Plasmodium* and *Toxoplasma*. Indications for clonal evolution are present in some populations. One *Cr. hominis* MLG is dominant and widespread in the UK [59]. Some *Cr. andersoni* MLGs are widespread in North America and the Czech Republic [60] and in several Chinese regions [61]. LD evidence is strong in *Cr. hominis* [7], [59], [62] and *Cr. parvum* [9], [59]. However, the impact of the Wahlund effect was not taken into account in [7], [62]. Near-clading can be suspected in *Cr. hominis* [7], *Cr. parvum* [13], and *Cr. muris* [61], although the evidence is less clear than for *Giardia* and the CNC. Lastly, panmixia was inferred in some populations of *Cr. parvum* [54], [55]. It is possible that *Cryptosporidium* population structure is similar to that of *P. falciparum* and *P. vivax* [11], with a continuum between panmixia and clonality and the existence of unstable near-clades. As for *Plasmodium*, whether clonality is due to starving sex or in-built genetic properties should be explored in depth. Obviously, the issue of *Cryptosporidium* population structure deserves further investigation.

Lastly, some indications for clonality were found in *Pn. jirovecii* [45]. However, evidence is far too limited to reach any firm conclusions.

## Implications for Molecular Epidemiology and Experimental Evolution

LD permits indirect typing; that is to say, the characterization of whole genotypes with only one gene, or a few genes. When LD is doubtful, indirect typing can be grossly misleading. This could be the case for *Cryptosporidium* subtyping with the unique gp60 gene [63]. If recombination is frequent, multilocus typing [64] is not a solution since frequent recombination makes the MLGs ephemeral. Still, the fact remains that the population structure of *Cryptosporidium* is far from being panmictic. Even if it is not strong enough to lead to stable near-clades, restrained recombination in these parasites constitutes a major stratification factor that should be taken into account in molecular epidemiology and all applied studies, as it should in *Plasmodium* [11].

When the evidence for PCE is clear, clonal MLGs and near-clades are convenient units of analysis for both molecular epidemiology and experimental evolution [3], thanks to their stability in space and time. Near-clades can be characterized by specific markers [13].

## Taxonomical Implications

We have called attention to the fact that radically dissimilar taxonomical inferences could be drawn from similar sets of data [65]. Scientists working on the pathogens here surveyed have granted considerable attention to taxonomical problems and species definition and delimitation. The conclusions they have reached vary considerably. The PCE model allows reconsidering these questions.

Two main species concepts are involved in these debates: the biological species concept (BSC) [66] and the phylogenetic species concept (PSC) [67]. The BSC demands two criteria: (i) within the species, genetic flow should have no other limitations than physical obstacles (potential panmixia) and (ii) it should be inhibited between species by built-in biological mechanisms. The PSC stipulates that species should correspond to clades, between which, by definition, gene flow is interrupted. Generally, authors propose a mix of genetic and biological characteristics to define species [68]. Some attempts have been made to apply the BSC concept to the CNC: experiments have shown that crosses within *C. gattii* VG II are easy, while they are difficult between II and III [31]. The authors have proposed that II and III deserve the status of biological species. This is debatable for two reasons: (i) experiments tell nothing about the frequency of recombination in nature [3] and (ii) the presence of stable genetic subdivisions (Russian doll near-clades) in VG II [31], [42] clearly shows that VG II is not a potentially panmictic entity. Also, by the survey of natural populations, it has been proposed [5] to equate the CNC “genotypic groups” to biological species. Nevertheless, as shown above, many PCE manifestations are observed within these groups.

The BSC has been proposed for the *Cryptosporidum* species [64], although, as we have seen above, recombination is restrained in some populations of this parasite.

Attributing the species status to the *Giardia* assemblages still is a matter of debate [4], [6], [16], [69], [70].

Lastly, as we have seen, the host-specific *Pneumocystis* genotypes are now considered as distinct species, although they could be equated, as well, to near-clades.

We propose that the BSC is not applicable to most, if not all, micropathogens. First, even between different species, very often, some genetic exchange occurs. Second, more importantly, clonality occurring in many populations of micropathogens makes it impossible to consider them as potentially panmictic units.

The PCE concept, and more specifically, the near-clade and Russian doll models, give an opportunity to apply the PSC to most pathogen species. The flexible phylogenetic approach based on the congruence principle relaxes the demands of a strict cladistic approach. The near-clades can be the starting units (necessary, but not sufficient) for species description based on the PSC adapted to the special case of micropathogens (lack of strictly separated intraspecific clades). It would then be the decision of specialists working on the considered pathogen to decide whether the specific biological properties and medical relevance of the near-clades (host specificity, pathogenicity, and drug resistance) justify that they be described as new species.

## Conclusion

We have provided clear evidence that the PCE model as it is formulated in the present study is verified in many pathogens, including viruses, bacteria, parasitic protozoa, and fungi [1]–[3], [10], [11]. The PCE model provides a convenient population genetics framework for all applied studies (strain typing, vaccine and drug design, and molecular and immunological diagnosis) dealing with the pathogens here surveyed and for experimental evolution. As a matter of fact, it provides these studies with stable, clearly defined units of analysis (clonal MLGs, near-clades). Moreover, it might bring a renewal of the long-lasting controversies concerning the species status of *Cryptosporidium*, *Giardia*, *Cryptococcus*, and *Pneumocystis.*
